# Convenience versus Biological Significance: Are PMA-Differentiated THP-1 Cells a Reliable Substitute for Blood-Derived Macrophages When Studying *in Vitro* Polarization?

**DOI:** 10.3389/fphar.2018.00071

**Published:** 2018-02-22

**Authors:** Serena Tedesco, Federica De Majo, Jieun Kim, Annalisa Trenti, Lucia Trevisi, Gian Paolo Fadini, Chiara Bolego, Peter W. Zandstra, Andrea Cignarella, Libero Vitiello

**Affiliations:** ^1^Venetian Institute of Molecular Medicine, Padova, Italy; ^2^Department of Biology, University of Padova, Padova, Italy; ^3^Institute of Biomaterials and Biomedical Engineering, University of Toronto, Toronto, ON, Canada; ^4^Department of Pharmaceutical and Pharmacological Sciences, University of Padova, Padova, Italy; ^5^Department of Medicine, University of Padova, Padova, Italy; ^6^The Donnelly Centre for Cellular and Biomolecular Research, University of Toronto, Toronto, ON, Canada; ^7^Medicine by Design, University of Toronto, Toronto, ON, Canada; ^8^Interuniversity Institute of Myology (IIM), Italy

**Keywords:** macrophage activation, phagocytosis, phenotype, THP-1 macrophages, human macrophages, migration, progenitor cells

## Abstract

Human peripheral-blood monocytes are used as an established *in vitro* system for generating macrophages. For several reasons, monocytic cell lines such as THP-1 have been considered as a possible alternative. In view of their distinct developmental origins and phenotypic attributes, we set out to assess the extent to which human monocyte-derived macrophages (MDMs) and phorbol-12-myristate-13-acetate (PMA)-differentiated THP-1 cells were overlapping across a variety of responses to activating stimuli. Resting (M0) macrophages were polarized toward M1 or M2 phenotypes by 48-h incubation with LPS (1 μg/ml) and IFN-γ (10 ng/ml) or with IL-4 (20 ng/ml) and IL-13 (5 ng/ml), respectively. At the end of stimulation, MDMs displayed more pronounced changes in marker gene expression than THP-1. Upon assaying an array of 41 cytokines, chemokines and growth factors in conditioned media (CM) using the Luminex technology, secretion of 29 out of the 41 proteins was affected by polarized activation. While in 12 of them THP-1 and MDM showed comparable trends, for the remaining 17 proteins their responses to activating stimuli did markedly differ. Quantitative comparison for selected analytes confirmed this pattern. In terms of phenotypic activation markers, measured by flow cytometry, M1 response was similar but the established MDM M2 marker CD163 was undetectable in THP-1 cells. In a beads-based assay, MDM activation did not induce significant changes, whereas M2 activation of THP-1 decreased phagocytic activity compared to M0 and M1. In further biological activity tests, both MDM and THP-1 CM failed to affect proliferation of mouse myogenic progenitors, whereas they both reduced adipogenic differentiation of mouse fibro-adipogenic progenitor cells (M2 to a lesser extent than M1 and M0). Finally, migration of human umbilical vein endothelial cells was enhanced by CM irrespective of cell type and activation state except for M0 CM from MDMs. In summary, PMA-differentiated THP-1 macrophages did not entirely reproduce the response spectrum of primary MDMs to activating stimuli. We suggest that THP-1 be regarded as a simplified model of human macrophages when investigating relatively straightforward biological processes, such as polarization and its functional implications, but not as an alternative source in more comprehensive immunopharmacology and drug screening programs.

## Introduction

Bone marrow–derived monocytes give rise to macrophages in some tissues, as well as during acute infection and inflammation (Geissmann et al., 2010; Hettinger et al., 2013; Wynn et al., 2013). Macrophages are a heterogeneous and plastic cell population which, by integrating signals emanating from the environment, can be activated into a spectrum of phenotypes ranging from the pro-inflammatory classically activated (M1) to anti-inflammatory alternatively activated macrophages (M2) (Sica and Mantovani, 2012; Murray et al., 2014; Xue et al., 2014). Dynamic changes in their activation states may also occur, and the final phenotype depends on the tissue in which they are found (e.g., osteoclasts, alveolar macrophages, Kupffer cells) as well as on their specific function (e.g., M1 or M2 macrophages, tumor-associated macrophages; Davies et al., 2013). Nonetheless, differentiated macrophages retain expression of a number of species-specific surface markers such as CD11b, F4/80, CD68 and CD163 (Gordon et al., 2014).

When it comes to investigating the pathways involved in macrophage activation and their pharmacological control, the source of macrophages, the definition of the activators, and the choice of surface markers and transcriptional regulation used to describe the type of activation are critical factors (Murray et al., 2014). A number of systematic validations of human macrophage phenotypic markers, as well as of maturation and activation methods, have recently been reported (Ambarus et al., 2012; Vogel et al., 2014; Tedesco et al., 2015). Surface markers are known to predict functional properties: for instance, CD206 expression allows prospective identification of phagocytic macrophages (A-Gonzalez et al., 2017). In addition, cell-cell interaction is important in terms of macrophage function in disease progression and tissue homeostasis. For instance, macrophage-derived factors are known to affect, among others, response to infection, resolution of inflammation, adipose tissue biology and cancer progression (Sica and Mantovani, 2012; Wynn et al., 2013).

To overcome the issues of limited lifespan and inter-individual variability that affects monocyte-derived human macrophages, the THP-1 acute monocytic leukemia cell line is frequently used in different research areas (Chanput et al., 2014). Following differentiation using phorbol 12-myristate 13-acetate (PMA) or other stimuli, THP-1 cells acquire a macrophage-like phenotype, which mimics primary human macrophages in several respects (Maeß et al., 2014; Lund et al., 2016). However, the malignant background of THP-1 cells might entail different responses compared to primary somatic cells in their natural environment. Hence, due to their distinct developmental origins and phenotypic attributes, the two cell models may not be overlapping across the full response spectrum, including cross-talk with other cell types.

Although differentiation and selected functional features of THP-1 cells were previously examined as compared to monocyte-derived macrophages (MDM; Kohro et al., 2004; Daigneault et al., 2010; Shiratori et al., 2017), to the best of our knowledge an extensive comparison of the two cell types in terms of potential crosstalk with other cell types *via* secreted factors following activation with pro- or anti-inflammatory stimuli has not been carried out yet. Hence, we set out to investigate the responses of THP-1-derived and human MDMs to M1- or M2-associated stimuli using a variety of experimental assays. In particular, besides analyzing the transcriptional and secretional profiles of both cell types, we chose to investigate the effect of their CM on three cell populations: satellite cells, fibroadipogenic progenitors and endothelial cells. The first two are main players in the repair and regeneration of skeletal muscle, a process in which macrophages play a paramount role (Juban and Chazaud, 2017); the third is instead involved in a wide range of physiological and pathological processes, from tissue repair to cancer growth in tight relationship with the inflammatory responses. The output of these analyses may be relevant to cell model selection for several applications, such as immunopharmacology studies and drug screening programs.

## Materials and Methods

### Cell Cultures

#### THP-1

THP-1 cells (ATCC^®^ TIB-202^TM^) were purchased from the American Type Culture Collection and cultured according to their specific indications, using an RPMI 1640 medium supplemented with non-heat-treated 10% fetal bovine serum (FBS; Invitrogen), 2 mM L-glutamine, 0.05 mM β-mercaptoethanol, 10 mM HEPES, 4500 mg/L glucose, 100 U/ml penicillin and 100 μg/ml streptomycin at 37°C in a humidified 5% CO_2_ atmosphere. THP-1 cells were kept at a minimum density of 3 × 10^5^ cells/ml and were passaged when reaching 8 × 10^5^ cells/ml. Upon thawing, cells were initially expanded by adding a volume of fresh medium every 48 h until they reached the above-mentioned maximum density, after which they were passaged every 2 days with a complete medium replacement.

#### Human Monocyte-Derived Macrophages (MDM)

Blood was obtained from male, non-smoking healthy donors aged 18–35, at the University of Padua Medical Center Transfusion Unit, following institutional standard operating procedures. PBMCs from buffy coats were isolated first by Ficoll-Paque (GE Healthcare) density gradient centrifugation at 400 *g* for 25 min followed by a second, high-density hyperosmotic Percoll gradient (GE Healthcare) at 400 *g* for 15 min. Monocytes were then seeded at 5 × 10^5^/ml in RPMI 1640 medium supplemented with 10% FBS (Invitrogen), 100 U/ml penicillin and 100 μg/ml streptomycin in the presence of 20 nM CSF-1 (Repnik et al., 2003). Cells were cultured for 7 days at 37°C and 5% CO_2_, with medium change every 3 days, to obtain MDMs.

#### Satellite Cells

Satellite cells were isolated from single myofibers of extensor digitorum longus (EDL) muscles of C57BL/10ScSn mice following a standard protocol (Pasut et al., 2013). Cells were expanded on gelatine-coated cell plates in F12 nutrient mixture (Ham) supplemented with 20% FBS (Gibco), 5 ng/ml FGFb, 100 U/ml penicillin and 100 μg/ml streptomycin, at 37°C in a humidified 5% CO_2_ atmosphere. Experiments were performed on cells with less than 10 passages.

#### Fibro-Adipogenic Precursors (FAPs)

Fibro-adipogenic precursors (kindly provided by Dr. Luca Madaro) were isolated by sorting from adult murine skeletal muscles, as CD45^-^CD31^-^ter119^-^α7int^-^sca1^+^. Cells were expanded in DMEM supplemented with 20% FBS (Gibco), 10% HS (Gibco), 2.5 ng/ml FGFb, 100 U/ml penicillin and 100 μg/ml streptomycin, on gelatin-coated cell plates. For adipogenic differentiation proliferating medium was replaced by DMEM supplemented with 10% FBS (Gibco), 0.25 μM dexamethasone, 0.5 mM 3-isobutyl-1-methylxanthine (IBMX), 10 μg/ml insulin, 100 U/ml penicillin and 100 μg/ml streptomycin. After 3 days in differentiation medium cells were exposed to an adipogenic maintenance medium DMEM supplemented with 10% FBS (Gibco), 10 μg/ml insulin, 100 U/ml penicillin and 100 μg/ml streptomycin.

### Human Umbilical Vein Endothelial Cells (HUVECs)

Human umbilical vein endothelial cells were isolated from normal-term umbilical cords as previously published (Bolego et al., 2006). Cells were grown in medium M199 (Invitrogen) supplemented with 15% FBS (Invitrogen), 40 μg/ml gentamicin, endothelial cell growth factor (ECGF, 100 μg/ml; Sigma–Aldrich), and heparin (100 UI/ml, Sigma–Aldrich), at 37°C in a humidified 5% CO_2_ atmosphere. HUVECs were identified by their morphology and detection of CD31-related antigen, and used for experiments from passages 2 through 5.

### Preparation of Conditioned Media (CM)

#### THP-1

Cell differentiation was induced via a 6-h exposure to 185 ng/ml phorbol 12-myristate 13-acetate (PMA, Sigma–Aldrich) in DMSO. Cells were then polarized toward M1 or M2 phenotype by incubation for 48 more hours with INF-γ (20 ng/ml, Immunotools) and LPS (100 ng/ml, Sigma–Aldrich) or with IL-4 (20 ng/ml, Immunotools) and IL-13 (20 ng/ml, Immunotools), respectively (Tjiu et al., 2009; Maeß et al., 2014), still in the presence of PMA. Cells used for the resting condition were kept in the presence of PMA for 48 more hours in normal growth medium. CM were then prepared by keeping polarized as well as resting cells in serum-free RPMI without stimuli and no PMA for 72 more hours. Media were then collected and concentrated 10-fold using Amicon Ultra-15 centrifugal filter units with Ultracel-PL, cut-off 3 KDa (Millipore/Merck). Total protein content was then determined by Bradford assay, using bovine serum albumin as reference. CM were stored at -20°C until use.

#### MDM

After removing the culture medium, resting macrophages were either incubated with fresh, serum-free RPMI to generate M0 or activated toward M1 or M2 phenotype by incubation for 48 h with either LPS (1 μg/ml, Sigma–Aldrich) and IFN-γ (10 ng/ml, Immunotools), or IL-4 (20 ng/ml, Immunotools) and IL-13 (5 ng/ml; Immunotools), respectively (Tedesco et al., 2015). To obtain CM, MDMs activated as described above were incubated for a further 72 h in serum-free RPMI without stimuli. Media were then collected and concentrated as described above for THP-1.

### qRT Analyses

For qRT analyses, THP-1 cells and MDMs were lysed immediately after the 48-h polarization step using the TRIzol^®^ Reagent (Life Technologies); total RNA isolation was performed according to the manufacturer’s protocol. cDNA was generated using EuroScript Reverse Transcriptase (Euroclone Cytogenetics), with random examers and 2.5 μg RNA per reaction. qRT-PCR reactions were then prepared with the PowerUp SYBR Green Mix (Applied Biosystems) and run using a QuantStudio 6 Flex Real-Time PCR System (Applied Biosystems). Primer pairs were selected from PrimerBank (The Massachusetts General Hospital, Boston, MA, United States) or, when not available, designed *ex novo* with NCBI Primer-BLAST. Verification and location of target gene sequences were performed on Ensembl Genome Browser. All primer sequences are reported in **Table 1**. Results were normalized using GAPDH as housekeeping gene as reference and evaluated using the 2^-ΔΔCt^ method.

**Table 1:** Sequences of the primers used for qRT analyses.

Gene	Forward	Reverse	Primer Bank ID	Amplicon Size
IL-6	ACTCACCTCTTCAGAACGAATTG	CCATCTTTGGAAGGTTCAGGTTG	224831235c1	149
TNF-α	CCTCTCTCTAATCAGCCCTCTG	GAGGACCTGGGAGTAGATGAG	25952110c1	220
HIF1α	TGCTCATCAGTTGCCACTTC	CGGCATCCAGAAGTTTTCTC	n/a	107
IL-1β	ATGATGGCTTATTACAGTGGCAA	GTCGGAGATTCGTAGCTGGA	27894305c1	132
CD68	CTTCTCTCATTCCCCTATGGACA	GAAGGACACATTGTACTCCACC	n/a	105
MCP-1	GATCTCAGTGCAGAGGCTCG	TTTGCTTGTCCAGGTGGTCC	n/a	155
TLR2	GCTCGGAGTTCTCCCAGTTTC	GAGCTGCCCTTGCAGATAC	n/a	299
TLR4	CAGAGTTGCTTTCAATGGCATC	AGACTGTAATCAAGAACCTGGAGG	n/a	282
CD206 (MRC1)	CTACAAGGGATCGGGTTTATGGA	TTGGCATTGCCTAGTAGCGTA	145312260c3	105
PPARγ	TACTGTCGGTTTCAGAAATGCC	GTCAGCGGACTCTGGATTCAG	116284372c3	141
CD204 (MSR1)	CCAGGTCCAATAGGTCCTCC	CTGGCCTTCCGGCATATCC	n/a	94
TGM2	CGTGACCAACTACAACTCGG	CATCCACGACTCCACCCAG	n/a	136
CCL22 (MDC)	ATTACGTCCGTTACCGTCTG	TAGGCTCTTCATTGGCTCAG	n/a	175
IL-10	TACGGCGCTGTCATCGATTT	TAGAGTCGCCACCCTGATGT	n/a	191


*Primers were selected from PrimerBank or designed using the online tool for Real-Time PCR Blast, as described in Section “Materials and Methods.”*

### Luminex Assays

Composition of CM was analyzed using Eve Technologies’ Human Cytokine/Chemokine Array 41-Plex Discovery assay (Eve Technologies Corp, Calgary, AB, Canada); THP-1 media were analyzed with a modified version of the platform, Human Cytokine/Chemokine Array 42-Plex Discovery assay, which included IL-18. For these analyses, both THP-1 and MDM CM were diluted to a twofold concentration compared to straight medium (pilot tests were performed to find out the optimal dilution). Given that MDM and THP-1 CM were analyzed in different runs, a quantitative comparison for single analytes was performed only when the data fulfilled the following criteria: (a) the standard curves of the two data sets had overlapping shapes, and (b) the fluorescence intensity (FI) values obtained from all the samples of at least one cell type fell within the central part of the standard curve (i.e., were equal or higher than the third smallest standard).

### Flow Cytometry

Surface marker expression in resting (M0), M1 and M2-polarized THP-1 and MDM cells was analyzed by flow cytometry. After the 48-h polarization, cells were washed once with PBS, gently scraped and transferred into FACS tubes. Cells were then stained with fluorochrome-tagged monoclonal antibodies (all from BD Biosciences) against surface CD80 (FITC) to typify the M1 phenotype, and against CD206 (FITC) and CD163 (PE) to characterize the M2 phenotype. The selection of markers was based on previous characterizations (Fadini et al., 2013; Tedesco et al., 2015; Toniolo et al., 2015) according to recent guidelines (Murray et al., 2014). Upon labeling, cells were washed, suspended in PBS and analyzed with a FacsCanto II flow cytometer (BD Biosciences), recording at least 10,000 events for each sample. Data were analyzed using the FacsDiva software (BD Biosciences). Isotype-matched controls were used as baseline reference. Typically, less than 2% positive cells were allowed beyond the statistical marker in appropriate controls.

### Phagocytosis Assays

Phagocytosis functional assay was performed in resting, M1- and M2-polarized THP-1 cells and MDMs. After 48-h polarization, cells were incubated with fluorescent beads (1.0 μm carboxylate-modified yellow-green fluospheres, Molecular Probes) for 1 h in serum-free RPMI at 37°C, 5% CO_2_ (Schrijvers et al., 2004). Cells were then washed three times with cold PBS to remove fluorescent beads that had not been internalized. Finally, macrophages were scraped from the plate and analyzed by flow cytometry.

### Proliferation Assays

Satellite cells were seeded into 24-well plates containing gelatine-coated glass coverslips, at a density of 5000 cells/cm^2^ in growth medium. The specified CM were then added to three wells per experimental condition. The amount of conditioned medium added to each well was chosen so that the total amount of protein equalled that contained in J774 macrophage conditioned medium (mCM, prepared as described in Malerba et al., 2009) used at 2% v/v as a positive control. Cells were kept in the presence of the CM at least 48 h, before being exposed to a 12-h pulse of 10 μM EdU (5-ethynyl-2′-deoxyuridine). After labeling, cell proliferation was assessed with the Click-iT^®^ EdU Alexa Fluor^®^ 488 Imaging Kit (Life Technologies) according to the manufacturer’s protocol. Images for EdU-positive cells were acquired with an epifluorescence microscope and the percentage of labeled nuclei (i.e., of cells that had gone through S phase during the labeling pulse) was determined by counting at least 10 randomly chosen fields per sample.

### Adipogenesis Assays

Fibro-adipogenic precursors cells were grown and differentiated in 24-well plates as described above, adding the specified CM to three wells per each experimental condition. The amount of conditioned medium added to each well was chosen so that the total amount of protein equalled that contained in the mCM used at 2% v/v as positive control. Once the 3 days in adipogenic maintenance medium were completed, cultures were fixed overnight in 4% PFA in PBS and stained with a 0.2% Oil-Red O solution in 60% iso-propanol. Oil-red O positive cells were counted at the bright-field optical microscope, either in at least 10 randomly chosen different fields or in the whole well.

### Chemotaxis Assay

Chemotaxis experiments were performed in a 48-well modified Boyden chamber (Neuro Probe) using 8-μm Nucleopore polyvinylpyrrolidine-free polycarbonate filters coated with 10 μg/ml collagen as described elsewhere (Trenti et al., 2017). Lower chambers were filled with 28 μL CM or M199 supplemented with 100 U/mL heparin in the presence of 10 ng/mL VEGF as a positive control. Upper chambers were filled with 50 μL HUVEC (1.6 × 10^5^ cells/mL in M199 supplemented with 1% FBS and 100 U/mL heparin). For assessment of basal motility, M199 supplemented with 100 U/mL heparin was used in the lower chamber. After 6 h incubation at 37°C, non-migrating HUVECs in the upper surface of the filter were removed by scraping. Cells migrated to the lower side of the filter were stained with Diff-Quick stain (Medion Diagnostics), and densitometric analysis was performed using the Image J 1.47v software (NIH, United States). Six replicates were performed for each independent experiment.

### Statistical Analyses

Statistical analysis from at least 3 independent experiments was performed. Results are presented as mean values, with error bars representing the standard error (S.E.M.) of the average value. Throughout this work we chose to use non-parametric statistical tests, specifically, Kruskal–Wallis for variance analysis and Mann–Whitney for pairwise analysis, because of the elevated data dispersion found in our qRT and Luminex data sets. All tests were performed using the Prism 6 suite (GraphPad Software). Significance thresholds were chosen as described in detail in the Section “Results.”

## Results

### Analysis of Gene Expression upon M1 and M2 Polarization Shows More Pronounced Changes in MDM than in THP-1

**FIGURE 1:**
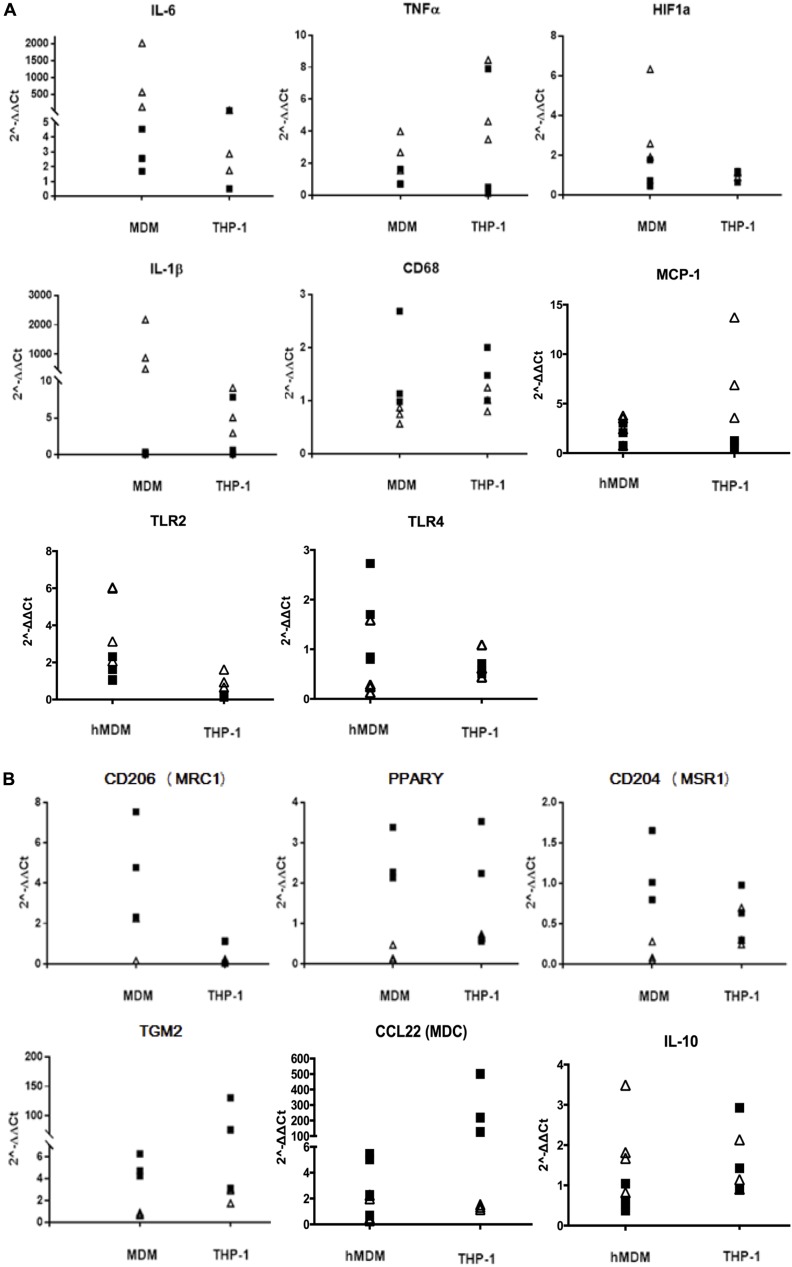
Gene expression profiles of macrophage activation markers. mRNA levels of M1 markers **(A)** and M2 markers **(B)** were measured by qRT and normalized to GAPDH. White triangles indicate the values found in M1-polarized cells; black squares indicate the values found in M2-polarized cells. Data are expressed as 2^-ΔΔCt^ values using the Resting condition as reference (*n* = 3–4 independent experiments per condition).

Differentiated MDM and THP-1 were activated for 48 h with either LPS/IFN-γ (M1) or IL-4/IL-13 (M2). Gene expression for a set of validated immunophenotypic markers for the two polarization states (Ambarus et al., 2012; Vogel et al., 2014; Chanput et al., 2014; Shiratori et al., 2017) was then tested by qRT-PCR with the 2^-ΔΔCt^ method, using the unpolarized (resting) condition as a reference. Upon M1 activation, mRNA levels of IL-6, HIF-α, IL-1β and TLR2 were significantly increased in MDM only, while MCP-1 did so in THP-1 cells only. No significant difference was found between M1 and M2 in either cell type for TNF-α, which tends to peak at earlier time points (Shiratori et al., 2017), and TLR4, whereas CD68 mRNA levels increased more in M2 than in M1, but once again only in MDM (**Figure 1A** and **Table 2**). Following M2 activation, mRNA levels of CD206, CD204 and PPARγ were significantly increased in MDM but not in THP-1, whereas TGM2 mRNA levels increased significantly in both cell types, CCL22/MDC increased significantly in THP-1 only, and IL-10 was unchanged in both groups (**Figure 1B** and **Table 2**).

**Table 2:** Statistical analysis of qRT data, performed using the Mann–Whitney non-parametric test.

	MDM		THP-1	
IL-6	^∗^	M1 > M2	ns	
TNF-α	ns		ns	
HIF-1α	^∗^	M1 > M2	ns	
IL-1β	^∗^	M1 > M2	ns	
TLR2	^∗^	M1 > M2	ns	
TLR4	ns		ns	
MCP-1	ns		^∗^	M1 > M2
MRC	^∗^	M2 > M1	ns	
PPAR-γ	^∗^	M2 > M1	ns	
CD68	^∗^	M2 > M1	ns	
CD204	^∗^	M2 > M1	ns	
TGM2	^∗^	M2 > M1	^∗^	M2 > M1
CCL22 (MDC)	ns		^∗^	M2 > M1
IL-10	ns		ns	


**^∗^0.1 ≥ p > 0.07, see Section “Results” for statistical considerations*.*

### Patterns of Cytokine Production upon Activation Exhibit Significant Differences between MDM and THP-1 Macrophages

**FIGURE 2:**
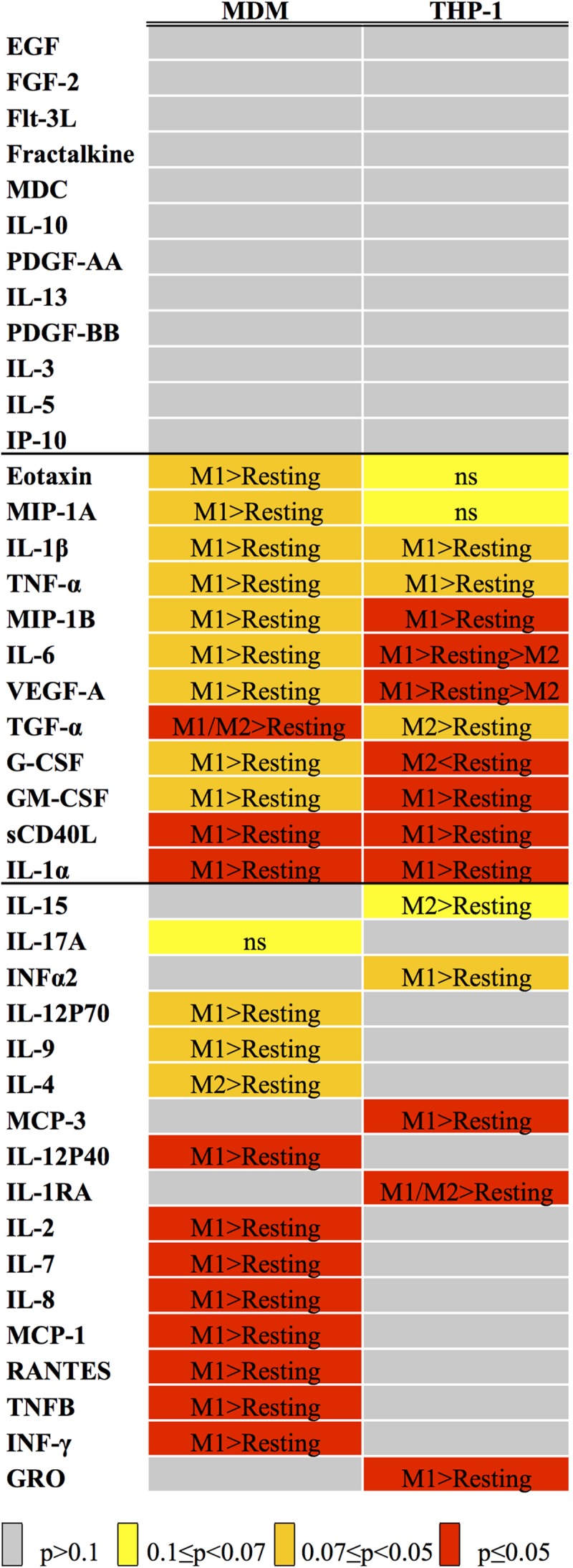
Statistical analysis of Luminex data. The heat map indicates the results of the variance analysis performed with the Kruskal–Wallis test considering the three experimental conditions (Resting, M1 and M2), expressed as *p*-values. The color code is listed below the map. When *p* was ≤0.1, we carried out a *post hoc* analysis with the Mann–Whitney test, comparing M1 versus Resting and M2 versus Resting. The results of such analyses are reported inside each cell (see Supplementary Table 1 for *p*-values).

The levels of 41 biologically active molecules (cytokines, chemokines and growth factors, **Table 3**) were measured in MDM and THP-1 conditioned supernatants using the Luminex technology; results are summarized in **Figure 2**. In 12 instances, polarization did not affect the protein’s secretion in either cell type (upper gray fields, **Figure 2**). In other 12 instances, THP-1 and MDM showed the same type of response to polarization (middle fields, **Figure 2**). By contrast, in the 17 remaining cases THP-1 and MDM differed in their responses to the polarization stimuli (lower fields, **Figure 2**), although just at the *p* > 0.07 level for IL-15 and IL-17A. It should be noticed that both cell types displayed a high data dispersion, which was reflected in high standard errors when averaging the independent experiments. For this reason, when assessing statistical significance, we considered a range of *p*-values rather than a single cut-off. Indeed, had we used a single significance threshold, even obvious biological differences would have not been acknowledged by our non-parametric analyses, as clearly demonstrated by the example of IL-1β (Supplementary Figure 1).

**Table 3:** Analytes evaluated with the Luminex assay.

M1-associated		M2-associated	Others
IL-1A	IP-10	IL-1RA	EGF
IL-1B	MCP-1	IL-4	Eotaxin
IL-2	MCP-3	IL-10	Flt-3
IL-3	MIP-1A	IL-12p40	FGF-2
IL-5	MIP-1B	IL-13	MDC
IL-6	G-CSF	TGF-α	PDGF-AA
IL-7	GM-CSF		PDGF-BB
IL-8	GRO		
IL-9	Fractalkine		
IL-12p70	RANTES		
IL-15	TNF-α		
IL-17A	TNF-β		
INFα2	VEGF-A		
INFγ			


**Cytokines and chemokines known to be preferentially secreted by a specific macrophage phenotype are indicated as M1 or M2*.*

MDM and THP-1 CM were also compared quantitatively, for those analytes in which the measurements fell within the requirements needed to compare datasets obtained in different runs (see Materials and Methods). The results of such analyses are shown in **Table 4**. No consistent trend was seen in terms of one cell type consistently producing higher amounts of active molecules compared to the other, although THP-1 media tended to come on top most of the time. The differences in expression levels were also quite variable, as they ranged from a minimum of twofold to almost three orders of magnitude.

**Table 4:** Quantitative comparison of Luminex analytes between MDM and THP-1.

Cytokine	*p*-value	Differences in		Log_2_ FC
TGF-α	^∗^	MDM < THP-1	Resting, M1, M2	3.9, 1.5, 3.3
MCP-3	^∗^	MDM > THP-1	M1	2.3
MDC	^∗^	MDM > THP-1	M1	2.3
PDGF-AA	^∗^	MDM < THP-1	Resting, M1, M2	8.6, 8.7, 8.5
PDGF-BB	ns	
IL-1RA	^∗^	MDM < THP-1	Resting, M1, M2	5.6, 6.1, 6.6
IL-1β	^∗^	MDM < THP-1	M1	6.5
IL-4	ns			
IL-6	^∗^	MDM > THP-1	M1	6.4
IL-8	^∗^	MDM < THP-1	Resting, M2	1.7, 3.0
IP-10	ns		
MCP-1	^∗^	MDM < THP-1	Resting, M2	1.1, 1.6
MIP-1A	^∗^	MDM < THP-1	Resting	2.8
MIP-1B	^∗^	MDM < THP-1	Resting, M1, M2	4.6, 2.6, 5.4
RANTES	^∗^	MDM < THP-1	Resting, M1, M2	4.4, 1.4, 3.3
TNF-α	^∗^	MDM < THP-1	M1	4.8
VEGF-A	^∗^	MDM < THP-1	Resting, M1, M2	6.4, 4.8, 5.4


**For each analyte, pairwise comparisons were performed between MDM/Resting versus THP-1/Resting, MDM/M1 versus THP-1/M1 and MDM/M2 versus THP-1/M2, using the Mann–Whitney non-parametric analysis. ^∗^Indicates 0.1 ≥ p > 0.07. The third column indicates which cell type had the highest secretion levels, as well as in which polarization state such difference was found. The last column shows the fold change for each of the conditions, expressed as base-2 logarithm*.*

### Expression of M2 Phenotypic Activation Markers in THP-1 Macrophages Does Not Match That Found in MDM

**FIGURE 3:**
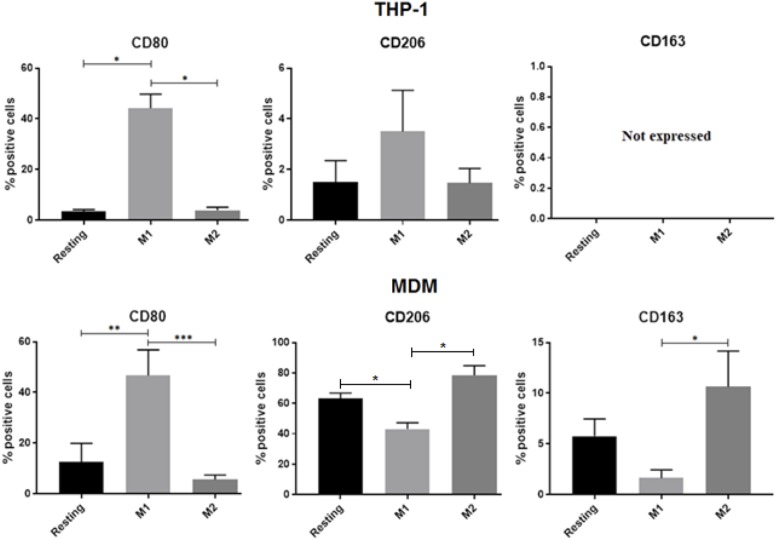
Flow cytometry analyses of surface activation markers for MDM and THP-1. Cells were unstimulated (resting) or activated with LPS/IFNγ or IL-4/IL-13 for 48 h. Bar graphs report the percentage of CD80^+^, CD206^+^, and CD163^+^ cells in 3 independent experiments with THP-1 macrophages **(A)** and in MDMs obtained from 4 different donors **(B)**. Data are expressed as mean ± SEM. Variance analysis was performed using the Kruskal–Wallis test and the *post hoc* analysis with the Mann–Whitney test. ^∗^0.1 ≥*p* > 0.07, ^∗∗^0.07 ≥*p* ≥ 0.05, ^∗∗∗^*p* ≤ 0.05.

Previous studies described marker expression in differently activated MDM (Ambarus et al., 2012; Vogel et al., 2014; Tedesco et al., 2015) and THP-1 cells (Chanput et al., 2014; Shiratori et al., 2017). Here we compared responses of three widely accepted polarization markers (i.e., CD80, CD206, and CD163) upon exposing the two cell types to the same polarization protocol. While response for the M1 marker CD80 was quite clear in both cell types (**Figure 3**), the M2 marker CD206 did not behave in the expected fashion in THP-1 cells. Specifically, its expression not only was in general confined to less than 4% of the cells but there was no increase in M2 compared to M1, as opposed to what was found in MDM. Furthermore, the M2 marker CD163, which also displayed the expected trend in MDM, was completely undetectable in THP-1 macrophages (**Figure 3**).

### THP-1 Macrophages Can Reproduce the Effects of MDM in Some Functional Assays, But Not in Others

**FIGURE 4:**
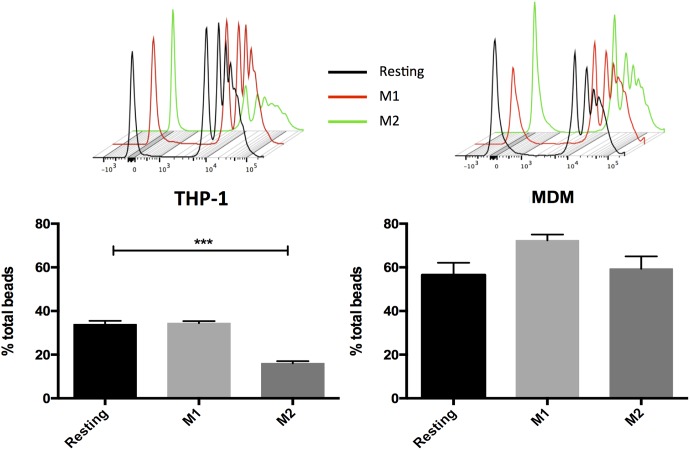
Macrophage phagocytic activity following incubation with dextran-FITC 1 μm beads. MDM and THP-1 were unstimulated (resting) or activated with LPS/IFNγ or IL-4/IL-13 for 48 h then incubated with fluorescent microbeads for 1 h. Data are expressed as mean ± SEM of 3 independent experiments with THP-1 and of 7 different donors for MDMs. Variance analysis was performed with the Kruskal–Wallis test and the *post hoc* analysis with the Mann–Whitney test. ^∗∗∗^*p* ≤ 0.05.

Phagocytosis is a major function of macrophages (Gordon, 2016). Using a beads ingestion assay, MDMs generally showed increased phagocytosis capacity as compared with THP-1. Activation of MDMs led to non-significant changes (see also Shiratori et al., 2017), whereas in THP-1 cells activation with IL-4/IL-13, but not with LPS/IFN-γ, downregulated the amount of internalized beads compared with resting (**Figure 4**), possibly as a result of limited amounts of cell surface M2 markers (**Figure 3**).

**FIGURE 5:**
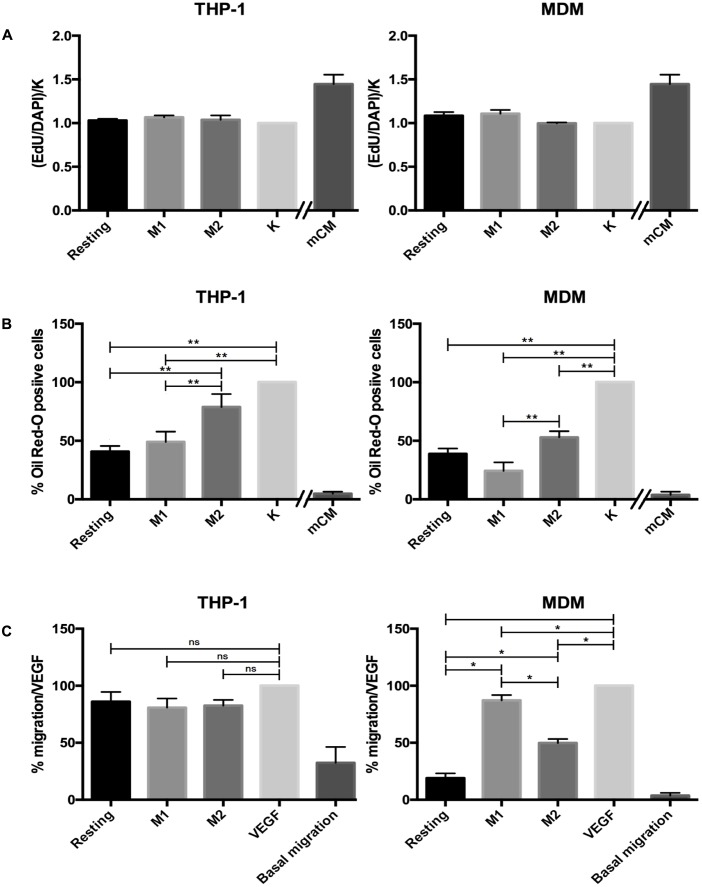
Results of functional assays. **(A)** Effect of THP-1 and MDM CM on satellite cells proliferation. The number of proliferating cells is expressed as the ratio of EdU positive cells in the different experimental conditions, normalized against the value found in non-treated cells (‘K’ column). Data are reported as mean ± SEM of 4 independent experiments with THP-1 and 5 independent experiments with MDM CM. mCM was used as positive control. **(B)** Effect of THP-1 and MDM CM on FAP adipogenesis. Extent of FAP adipogenesis is expressed as the fraction of Oil Red-O stained cells, normalized against the value found in non-treated cells (‘K’ column). Data are expressed as mean ± SEM of 7 independent experiments with THP-1 cells and 4 independent experiments with MDMs. mCM was used as positive control. **(C)** THP-1 and MDM CM influenced HUVEC migration. HUVEC migration was measured in a Boyden micro-chemotaxis chamber after 6 h. VEGF was used as a positive control. Data are expressed as mean ± SEM of 3 independent experiments for THP-1 and of 4 different donors for MDM, all performed in sextuplicate. Variance analysis was performed with the Kruskal–Wallis test and the *post hoc* analysis with the Mann–Whitney test. ^∗^0.1 ≥*p* > 0.07, ^∗∗^0.07 ≥*p* ≥ 0.05.

Several lines of evidence indicate that macrophages play an active role in controlling proliferation and differentiation in the context of skeletal muscle regeneration (Juban and Chazaud, 2017). For this reason, we assessed the effect of conditioned MDM versus THP-1 media on the proliferation of murine satellite cell-derived myogenic progenitors. Satellite cells are adult myogenic stem cells that are located under the basal lamina surrounding each myofiber and are responsible for muscle regeneration in response to injury. This model was chosen because our data had indicated that mCM from LPS-activated J774 cells had powerful pro-myogenic effects both *in vitro* and *in vivo* (Malerba et al., 2009 and *manuscript in preparation*). As shown in **Figure 5A**, neither THP-1 nor MDM media affected the proliferation rate, while the mCM increased the fraction of EdU^+^ cells, as expected.

The second cellular model onto which we tested THP-1 and MDM CM was the differentiation of muscle-derived FAPs. These are also cells involved in muscle regeneration, as upon damage FAPs release trophic factors acting on satellite cells, but in pathological conditions their over-activation can lead to the typical fibro-fatty deposition found in many muscle diseases. Once again, we used mCM as positive control, as it has a potent anti-adipogenic effect (*manuscript in preparation*). Exposure of FAPs to all three THP-1 CM reduced adipogenic differentiation, measured as % of Oil Red O-stained cells (**Figure 5B** and Supplementary Figure 3); such effect was more evident with resting and M1 than with M2. The same trend was observed with MDM media, except that M2 and resting media yielded comparable effects. At the same time, neither MDM nor THP-1 CM proved as potent as murine CM in their anti-adipogenic effect.

Lastly, we assessed chemotactic effects of THP-1 and MDM CM on human primary endothelial cells (HUVECs) using the micro-chemotaxis chamber assay. In these experiments, human VEGF was used as a positive control. In this model, the effect of MDM media differed greatly from that of THP-1, as the latter induced chemoattractant effects comparable to VEGF even in the resting state, with no changes in response to any activating stimuli. Conversely, in MDM only media from M1 and, to a lesser extent, M2 cells induced an increase in HUVEC migration, while no effect was seen with medium from resting cells (**Figure 5C**; see also Supplementary Figure 4 for an example of raw migration data).

## Discussion

Human peripheral-blood monocytes are the most commonly used precursors for generating macrophages *in vitro* (Murray et al., 2014). However, use of MDMs presents several issues, such as the fact that they are unable to proliferate to a significant extent (i.e., each prep requires a fresh supply of cells) and cannot be stored in liquid nitrogen. Besides, and this aspect can severely affect experimental data, donor-dependent variability can be substantial. Conversely, using the THP-1 cell line as a source for human macrophages does away with these issues, as they can be easily expanded *in vitro* and stocked in liquid nitrogen in their non-differentiated state, and their single genetic background should minimize the variability of cell phenotype (reviewed in Auwerx, 1991; Qin, 2012; Chanput et al., 2014).

THP-1 cells are derived from a tumor, however, and as such one cannot simply assume that the phenotypic and molecular attributes of the macrophages derived from their differentiation are necessarily equivalent to those of macrophages obtained from circulating monocytes. For example, as opposed to primary monocytes, monocyte-derived THP-1 cells express low levels of CD14 (Bosshart and Heinzelmann, 2004), a membrane-associated protein that forms a highly sensitive LPS signaling complex with TLR4 and MD2 (Park et al., 2009), and decreases with macrophage differentiation (Steinbach and Thiele, 1994). Indeed, even though THP-1 derived macrophages are functional and can be activated, their responsiveness in terms of gene expression changes upon activation has been reported to be smaller than that of naïve primary macrophages (Maeß et al., 2014).

These considerations prompted us to carry out a systematic comparison between MDM and THP-1 derived macrophages in terms of gene expression, cytokine and chemokine secretion, surface markers and functional responses on other cell types, both in resting and activated conditions. We took advantage of previous studies to select suitable differentiation and activation protocols for THP-1 (Daigneault et al., 2010) and MDM (Tedesco et al., 2015). Our qPCR analyses on 14 markers of polarization confirmed that, at the transcription level, activation of THP-1 cells often led to a different, usually less pronounced response compared to MDM. Such a limited polarization response of THP-1 in comparison to MDM is consistent with a recently published study (Shiratori et al., 2017). For example, the difference between M1 and M2 THP-1 cells in terms of IL-1β mRNA levels did not reach statistical significance; however, IL-1β protein concentration in THP-1 M1 conditioned medium was much higher than that in M2 and resting media (**Figure 2** and Supplementary Figure 2). This finding is in agreement with other transcriptome/proteome comparisons of different macrophage models, which reported that very few molecules are regulated at both mRNA and protein levels across cell types and activation states (Martinez et al., 2013; Xue et al., 2014).

A comprehensive analysis of secreted cytokines and growth factors in resting and activated states showed distinct signatures in the two cell types. Specifically, our data indicate that many of the analytes such as monocyte chemotactic protein (MCP)-1, MCP-3 and IL-4 did not show the same response to polarization in the two cell types. For a subset of analytes we could reliably compare the amount of protein secreted by both cell types. Once again, MDM and THP-1 macrophages exhibited noticeable differences, as for the single factors the change in concentration ranged from 2.5 to more than 400-fold, usually in favor of THP-1 cells. For example, levels of secreted IL-1β were about 90-fold higher in THP-1 than in MDM media, in agreement with previously published data (Daigneault et al., 2010), likely due to more complex IL-1β processing and two-step secretion in MDMs (Netea et al., 2009). Secretion of TNF-α following stimulation with LPS was also remarkably higher in PMA-treated THP-1 in comparison to MDMs. In contrast, secretion of MCP-3, macrophage-derived chemokine (MDC) and IL-6 was more pronounced in MDMs in comparison to THP-1. Of note, the secreted amount of each of the above proteins was significantly upregulated by M1-associated stimuli (**Table 4**) in MDMs, which was somewhat unexpected for MDC, regarded as a M2 marker by other authors (Grotenhuis et al., 2013). These findings suggest that the two macrophage models are unlikely to be fully interchangeable when testing functional effects of their conditioned media on other cell types *in vitro*.

In terms of phenotypic surface markers, while M1 polarization led to the expected outcome in both cells types, M2 polarization did not seem to affect THP-1 macrophages. Specifically, not only CD206 expression did not differ between M1 and M2 cells, but there was a trend toward increased CD206 levels in M1 in comparison to M2 THP-1 macrophages. Furthermore, these latter did not express detectable amounts of the hemoglobin receptor CD163, an established M2 marker regulated, among others, by glucocorticoids (Vallelian et al., 2010; Tedesco et al., 2015). Such limited response to M2 polarization has also been reported in a very recent study (Shiratori et al., 2017), in which, however, the Authors did report a basal expression of CD163, albeit unresponsive to polarization, both in MDM and in THP-1-derived macrophages.

Importantly, our set of functional assays also yielded mixed results in terms of equivalence of MDMs and THP-1. In the beads internalization assay, the phagocytic capacity of MDM was not modulated by M1- or M2-associated stimuli, whereas that of THP-1 macrophages was downregulated by IL-4/IL-13 activation. These findings are consistent with the view that phagocytosis is a general property of macrophages (Gordon, 2016), but not necessarily a reliable predictor of M1 or M2 responses (Mills and Ley, 2014). It should also be noted that the outcome of phagocytosis assays by macrophages can be affected by the phagocytosis-triggering agent, e.g., opsonised beads as opposed to bacterial antigens (Daigneault et al., 2010; Sumiya et al., 2015; Shiratori et al., 2017). In the second set of analyses, based on cells that are key players in skeletal muscle regeneration, MDM and THP-1 CM had comparable effects on satellite cells proliferation and FAPs adipogenic differentiation. Still, there was a difference involving THP-1 M2 macrophages, whose conditioned medium did not decrease FAP adipogenesis to a statistically significant extent. Finally, our chemotactic tests on HUVECs showed that migration was cell-type and activation-status-dependent, but there was a marked difference between MDM and THP-1 CM. Specifically, all three THP-1 CM induced HUVEC migration rate at a rate similar to that induced by VEGF alone. In contrast, CM from M1 and, to a lesser extent, M2 MDM positively regulated HUVEC migration compared with resting. These conclusions are in agreement with the results of our Luminex analyses, as IL-8, RANTES and VEGF-A, known to have chemoattractant effects on endothelial cells, were way more abundant in THP-1 than in MDM CM, independently from the activation state. Overall, our findings highlight the importance of testing the impact of macrophage polarized activation not only on functional changes in macrophages themselves, but also in other cell types mediated through phenotype- and macrophage model-specific signatures of secreted factors.

One limitation of this type of study is that any changes in experimental procedures such as the method used to cultivate THP-1 cells, the amount of glucose in culture media, criteria of changing medium, the selection of time points and the methods used for macrophage polarization (the latter being reviewed in Murray et al., 2014) might impact on several endpoints. However, previous studies using experimental conditions other than those used herein are consistent with the present findings (Daigneault et al., 2010; Spiller et al., 2016; Shiratori et al., 2017).

Altogether, our results indicate that THP-1 cells can indeed respond to the polarization protocols used for primary macrophages, but to an extent that can greatly vary depending on the specific endpoint, and that can either match or diverge from what is seen in human peripheral-blood MDMs. Therefore, THP-1 cell line reliability as an alternative model system to primary macrophages should not be taken for granted but rather confirmed whenever it is used in a new experimental setting. In addition, the output of these analyses may be relevant to cell model selection in immunopharmacology studies and drug screening programs.

## Author Contributions

PZ, AC, and LV were responsible for the concept and design of the study. ST, FDM, JK, and AT performed the experiments and contributed to data acquisition. ST, FDM, JK, LT, GF, CB, AC, and LV contributed to data analysis and interpretation. AC and LV drafted the manuscript. All authors were involved in revising and approved the final version of the manuscript.

## Conflict of Interest Statement

The authors declare that the research was conducted in the absence of any commercial or financial relationships that could be construed as a potential conflict of interest.

## References

[B1] A-Gonzalez N. Quintana J. A. García-Silva S. Mazariegos M. González de la Aleja A. Nicolás-Ávila J. A. (2017). Phagocytosis imprints heterogeneity in tissue-resident macrophages. *J. Exp. Med.* 214 1281–1296. 10.1084/jem.20161375 28432199 PMC5413334

[B2] Ambarus C. A. Krausz S. van Eijk M. Hamann J. Radstake T. R. Reedquist K. A. (2012). Systematic validation of specific phenotypic markers for in vitro polarized human macrophages. *J. Immunol. Methods* 375 196–206. 10.1016/j.jim.2011.10.013 22075274

[B3] Auwerx J. (1991). The human leukemia cell line, THP-1: a multifacetted model for the study of monocyte-macrophage differentiation. *Experientia* 47 22–31. 10.1007/BF02041244 1999239

[B4] Bolego C. Buccellati C. Radaelli T. Cetin I. Puglisi L. Folco G. (2006). eNOS, COX-2, and prostacyclin production are impaired in endothelial cells from diabetics. *Biochem. Biophys. Res. Commun.* 339 188–190. 10.1016/j.bbrc.2005.11.017 16297879

[B5] Bosshart H. Heinzelmann M. (2004). Lipopolysaccharide-mediated cell activation without rapid mobilization of cytosolic free calcium. *Mol. Immunol.* 41 1023–1028. 10.1016/j.molimm.2004.05.003 15302164

[B6] Chanput W. Mes J. J. Wichers H. J. (2014). THP-1 cell line: an in vitro cell model for immune modulation approach. *Int. Immunopharmacol.* 23 37–45. 10.1016/j.intimp.2014.08.002 25130606

[B7] Daigneault M. Preston J. A. Marriott H. M. Whyte M. K. Dockrell D. H. (2010). The identification of markers of macrophage differentiation in PMA-stimulated THP-1 cells and monocyte-derived macrophages. *PLoS One* 5:e8668. 10.1371/journal.pone.0008668 20084270 PMC2800192

[B8] Davies L. C. Jenkins S. J. Allen J. E. Taylor P. R. (2013). Tissue-resident macrophages. *Nat. Immunol.* 14 986–995. 10.1038/ni.2705 24048120 PMC4045180

[B9] Fadini G. P. de Kreutzenberg S. V. Boscaro E. Albiero M. Cappellari R. Kränkel N. (2013). An unbalanced monocyte polarisation in peripheral blood and bone marrow of patients with type 2 diabetes has an impact on microangiopathy. *Diabetologia* 56 1856–1866. 10.1007/s00125-013-2918-9 23616239

[B10] Geissmann F. Manz M. G. Jung S. Sieweke M. H. Merad M. Ley K. (2010). Development of monocytes, macrophages, and dendritic cells. *Science* 327 656–661. 10.1126/science.1178331 20133564 PMC2887389

[B11] Gordon S. (2016). Phagocytosis: an immunobiologic process. *Immunity* 44 463–475. 10.1016/j.immuni.2016.02.026 26982354

[B12] Gordon S. Plüddemann A. Martinez Estrada F. (2014). Macrophage heterogeneity in tissues: phenotypic diversity and functions. *Immunol. Rev.* 262 36–55. 10.1111/imr.12223 25319326 PMC4231239

[B13] Grotenhuis N. Bayon Y. Lange J. F. Van Osch G. J. Bastiaansen-Jenniskens Y. M. (2013). A culture model to analyze the acute biomaterial-dependent reaction of human primary macrophages. *Biochem. Biophys. Res. Commun.* 433 115–120. 10.1016/j.bbrc.2013.02.054 23485466

[B14] Hettinger J. Richards D. M. Hansson J. Barra M. M. Joschko A. C. Krijgsveld J. (2013). Origin of monocytes and macrophages in a committed progenitor. *Nat. Immunol.* 14 821–830. 10.1038/ni.2638 23812096

[B15] Juban G. Chazaud B. (2017). Metabolic regulation of macrophages during tissue repair: insights from skeletal muscle regeneration. *FEBS Lett.* 591 3007–3021. 10.1002/1873-3468.12703 28555751

[B16] Kohro T. Tanaka T. Murakami T. Wada Y. Aburatani H. Hamakubo T. (2004). A comparison of differences in the gene expression profiles of phorbol 12-myristate 13-acetate differentiated THP-1 cells and human monocyte-derived macrophage. *J. Atheroscler. Thromb.* 11 88–97. 10.5551/jat.11.88 15153668

[B17] Lund M. E. To J. O’Brien B. A. Donnelly S. (2016). The choice of phorbol 12-myristate 13-acetate differentiation protocol influences the response of THP-1 macrophages to a pro-inflammatory stimulus. *J. Immunol. Methods* 430 64–70. 10.1016/j.jim.2016.01.012 26826276

[B18] Maeß M. B. Wittig B. Cignarella A. Lorkowski S. (2014). Reduced PMA enhances the responsiveness of transfected THP-1 macrophages to polarizing stimuli. *J. Immunol. Methods* 402 76–81. 10.1016/j.jim.2013.11.006 24269601

[B19] Malerba A. Vitiello L. Segat D. Dazzo E. Frigo M. Scambi I. (2009). Selection of multipotent cells and enhanced muscle reconstruction by myogenic macrophage-secreted factors. *Exp. Cell Res.* 315 915–927. 10.1016/j.yexcr.2009.01.005 19371636

[B20] Martinez F. O. Helming L. Milde R. Varin A. Melgert B. N. Draijer C. (2013). Genetic programs expressed in resting and IL-4 alternatively activated mouse and human macrophages: similarities and differences. *Blood* 121 e57–e69. 10.1182/blood-2012-06-436212 23293084

[B21] Mills C. D. Ley K. (2014). M1 and M2 macrophages: the chicken and the egg of immunity. *J. Innate Immun.* 6 716–726. 10.1159/000364945 25138714 PMC4429858

[B22] Murray P. J. Allen J. E. Biswas S. K. Fisher E. A. Gilroy D. W. Goerdt S. (2014). Macrophage activation and polarization: nomenclature and experimental guidelines. *Immunity* 41 14–20. 10.1016/j.immuni.2014.06.008 25035950 PMC4123412

[B23] Netea M. G. Nold-Petry C. A. Nold M. F. Joosten L. A. Opitz B. van der Meer J. H. (2009). Differential requirement for the activation of the inflammasome for processing and release of IL- 1β in monocytes and macrophages. *Blood* 113 2324–2335. 10.1182/blood-2008-03-146720 19104081 PMC2652374

[B24] Park B. S. Song D. H. Kim H. M. Choi B. S. Lee H. Lee J. O. (2009). The structural basis of lipopolysaccharide recognition by the TLR4-MD-2 complex. *Nature* 458 1191–1195. 10.1038/nature07830 19252480

[B25] Pasut A. Jones A. E. Rudnicki M. A. (2013). Isolation and culture of individual myofibers and their satellite cells from adult skeletal muscle. *J. Vis. Exp.* 73:e50074. 10.3791/50074 23542587 PMC3639710

[B26] Qin Z. (2012). The use of THP-1 cells as a model for mimicking the function and regulation of monocytes and macrophages in the vasculature. *Atherosclerosis* 221 2–11. 10.1016/j.atherosclerosis.2011.09.003 21978918

[B27] Repnik U. Knezevic M. Jeras M. (2003). Simple and cost-effective isolation of monocytes from buffy coats. *J. Immunol. Methods* 278 283–292. 10.1016/S0022-1759(03)00231-X 12957415

[B28] Schrijvers D. M. Martinet W. De Meyer G. R. Andries L. Herman A. G. Kockx M. M. (2004). Flow cytometric evaluation of a model for phagocytosis of cells undergoing apoptosis. *J. Immunol. Methods* 287 101–108. 10.1016/j.jim.2004.01.013 15099759

[B29] Shiratori H. Feinweber C. Luckhardt S. Linke B. Resch E. Geisslinger G. (2017). THP-1 and human peripheral blood mononuclear cell-derived macrophages differ in their capacity to polarize in vitro. *Mol. Immunol.* 88 58–68. 10.1016/j.molimm.2017.05.027 28600970

[B30] Sica A. Mantovani A. (2012). Macrophage plasticity and polarization: in vivo veritas. *J. Clin. Invest.* 122 787–795. 10.1172/JCI59643 22378047 PMC3287223

[B31] Spiller K. L. Wrona E. A. Romero-Torres S. Pallotta I. Graney P. L. Witherel C. E. (2016). Differential gene expression in human, murine, and cell line-derived macrophages upon polarization. *Exp. Cell Res.* 347 1–13. 10.1016/j.yexcr.2015.10.017 26500109

[B32] Steinbach F. Thiele B. (1994). Phenotypic investigation of mononuclear phagocytes by flow cytometry. *J. Immunol. Methods* 174 109–122. 10.1016/0022-1759(94)90015-9 8083514

[B33] Sumiya Y. Ishikawa M. Inoue T. Inui T. Kuchiike D. Kubo K. (2015). Macrophage activation mechanisms in human monocytic cell line-derived macrophages. *Anticancer Res.* 35 4447–4451. 26168485

[B34] Tedesco S. Bolego C. Toniolo A. Nassi A. Fadini G. P. Locati M. (2015). Phenotypic activation and pharmacological outcomes of spontaneously differentiated human monocyte-derived macrophages. *Immunobiology* 220 545–554. 10.1016/j.imbio.2014.12.008 25582402

[B35] Tjiu J. W. Chen J. S. Shun C. T. Lin S. J. Liao Y. H. Chu C. Y. (2009). Tumor-associated macrophage-induced invasion and angiogenesis of human basal cell carcinoma cells by cyclooxygenase-2 induction. *J. Invest. Dermatol.* 129 1016–1025. 10.1038/jid.2008.310 18843292

[B36] Toniolo A. Fadini G. P. Tedesco S. Cappellari R. Vegeto E. Maggi A. (2015). Alternative activation of human macrophages is rescued by estrogen treatment in vitro and impaired by menopausal status. *J. Clin. Endocrinol. Metab.* 100 E50–E58. 10.1210/jc.2014-2751 25303489

[B37] Trenti A. Tedesco S. Boscaro C. Ferri N. Cignarella A. Trevisi L. (2017). The glycolytic enzyme PFKFB3 is involved in estrogen-mediated angiogenesis via GPER1. *J. Pharmacol. Exp. Ther.* 361 398–407. 10.1124/jpet.116.238212 28348059

[B38] Vallelian F. Schaer C. A. Kaempfer T. Gehrig P. Duerst E. Schoedon G. (2010). Glucocorticoid treatment skews human monocyte differentiation into a hemoglobin-clearance phenotype with enhanced heme-iron recycling and antioxidant capacity. *Blood* 116 5347–5356. 10.1182/blood-2010-04-277319 20739658

[B39] Vogel D. Y. Glim J. E. Stavenuiter A. W. Breur M. Heijnen P. Amor S. (2014). Human macrophage polarization in vitro: maturation and activation methods compared. *Immunobiology* 219 695–703. 10.1016/j.imbio.2014.05.002 24916404

[B40] Wynn T. A. Chawla A. Pollard J. W. (2013). Macrophage biology in development, homeostasis and disease. *Nature* 496 445–455. 10.1038/nature12034 23619691 PMC3725458

[B41] Xue J. Schmidt S. V. Sander J. Draffehn A. Krebs W. Quester I. (2014). Transcriptome-based network analysis reveals a spectrum model of human macrophage activation. *Immunity* 40 274–288. 10.1016/j.immuni.2014.01.006 24530056 PMC3991396

