# Supplementary material for: Convenience versus Biological Significance: Are PMA-Differentiated THP-1 Cells a Reliable Substitute for Blood-Derived Macrophages When Studying in Vitro Polarization?
Source: Front Pharmacol. 2018 Feb 22;9:71. doi: 10.3389/fphar.2018.00071 (PMC5826964; doi:10.3389/fphar.2018.00071)
Supplement: Supplementary file 1 [file Presentation_1.pdf]

	MDM				THP-1			
	M1		M2		M1		M2	
	p-value	Log_2 fold change *vs* Resting	p-value	Log_2 fold change *vs* Resting	p-value	Log_2 fold change *vs* Resting	p-value	Log_2 fold change *vs* Resting
Eotaxin	0.1	2.3	>0.1		>0.1		>0.1	
MIP-1A	0.1	3.2	>0.1		>0.1		>0.1	
IL-1β	0.1	3.7	>0.1		0.1	2.7	>0.1	
TNFa	0.1	2.0	>0.1		0.1	1.5	>0.1	
MIP-1B	0.1	3.5	>0.1		0.1	1.4	>0.1	
IL-6	0.1	4.3	>0.1		0.1	1.6	0.1	-2.6
VEGF-A	0.1	2.2	>0.1		0.1	0.6	0.1	-1.2
TGF-a	0.1	2.4	0.1	1.9	>0.1		0.1	1.3
G-CSF	0.1	3.0	>0.1		>0.1		0.1	-1.0
GM-CSF	0.1	2.0	>0.1		0.1	1.9	>0.1	
sCD40L	008	0.7	>0.1		0.1	1.1	>0.1	
IL-1a	0.1	7.3	>0.1		0.1	2.1	>0.1	
IL-15	na		na		>0.1		0.1	0.7
IL-17A	>0.1		>0.1		na		na	
INFa2	na		na		0.1	2.3	>0.1	
IL-12P70	0.08	1.8	>0.1		na		na	
IL-9	0.1	0.7	>0.1		na		na	
IL-4	>0.1		0.1	6.0	na		na	
MCP-3	na		na		0.1	1.7	>0.1	
IL-12P40	0.1	4.4	>0.1		na		na	
IL-1RA	na		na		0.1	0.7	0.1	0.9
IL-2	0.1	1.7	>0.1		na		na	
IL-7	0.1	1.7	>0.1		na		na	
IL-8	0.1	1.9	>0.1		na		na	
MCP-1	0.1	1.1	>0.1		na		na	
RANTES	0.1	3.3	>0.1		na		na	
TNFB	0.1	1.8	>0.1		na		na	
INFy	0.1	2.6	>0.1		na		na	
GRO	na		na		0.1	2.2	>0.1	

Supplementary Table 1. Statistical analysis of Luminex data. Results of the variance analysis performed with the Kruskal-Wallis test considering the three experimental conditions (Resting, M1 and M2) are expressed as *p* values. When *p* was ≤0.1, *post hoc* analysis was carried out using the Mann-Whitney test, comparing M1 *vs.* Resting and M2 *vs.* Resting. The results of such analyses are reported where indicated.

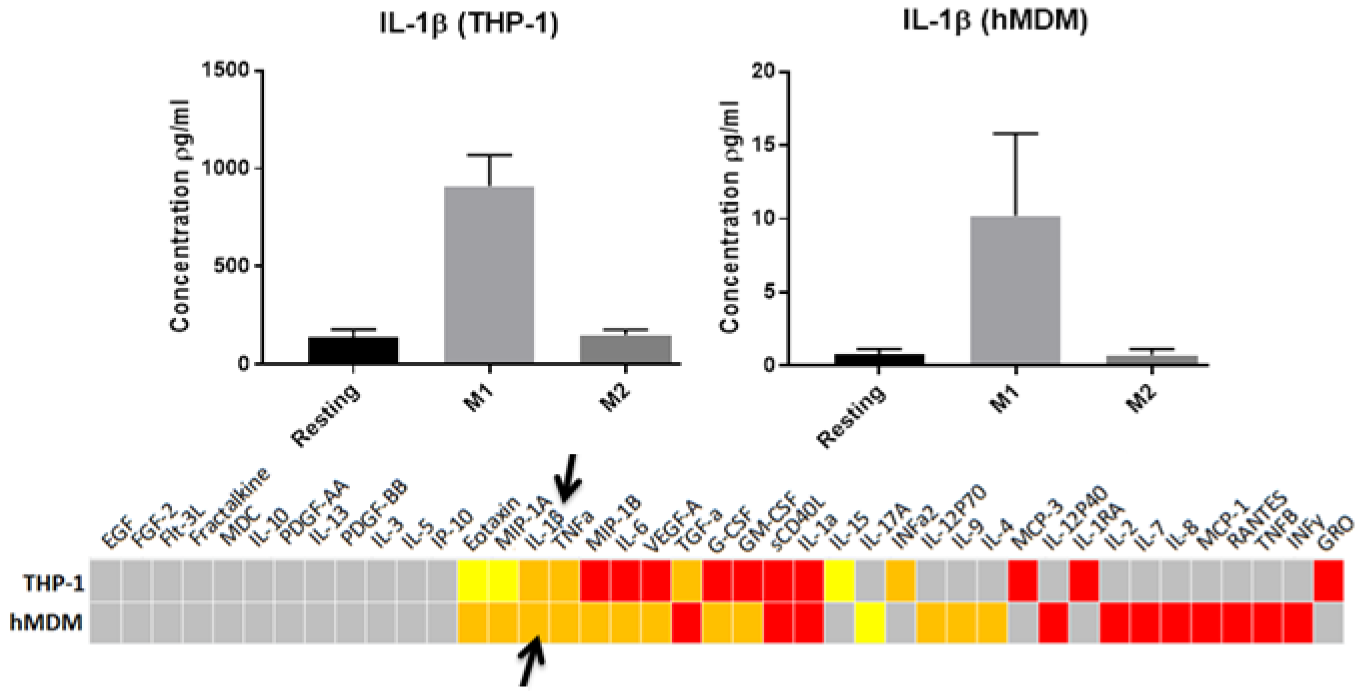

Supplementary Figure 1: Example of how setting a fixed cut-off at p=0.05 would have led, in many instances, to label as ‘non-significant’ clear biological differences in our data set. In the case of IL-1ß, a well-known M1 marker, M1 polarization leads to an obvious increase in secretion in both cell types. Still, variance analysis carried out with the Kruskal-Wallis test yielded p-values comprised between 0.07 and 0.05.

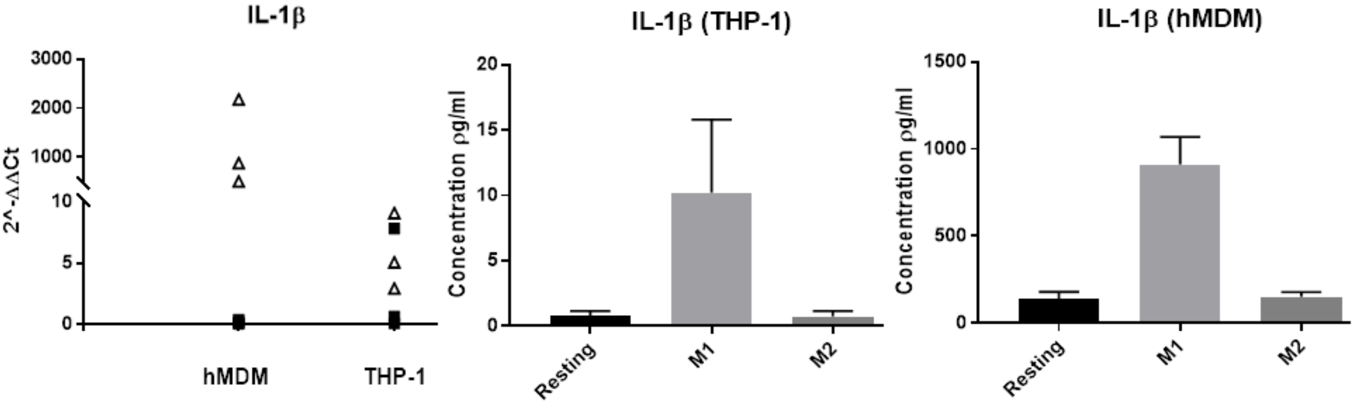

Supplementary Figure 2: In some instances, polarization affected gene expression and the protein secretion levels to different extents. Once again, taking IL-1β as an example, one can see how in THP-1 cells seemingly low differences at the transcriptional did not match the large variations found at the protein secretion level.

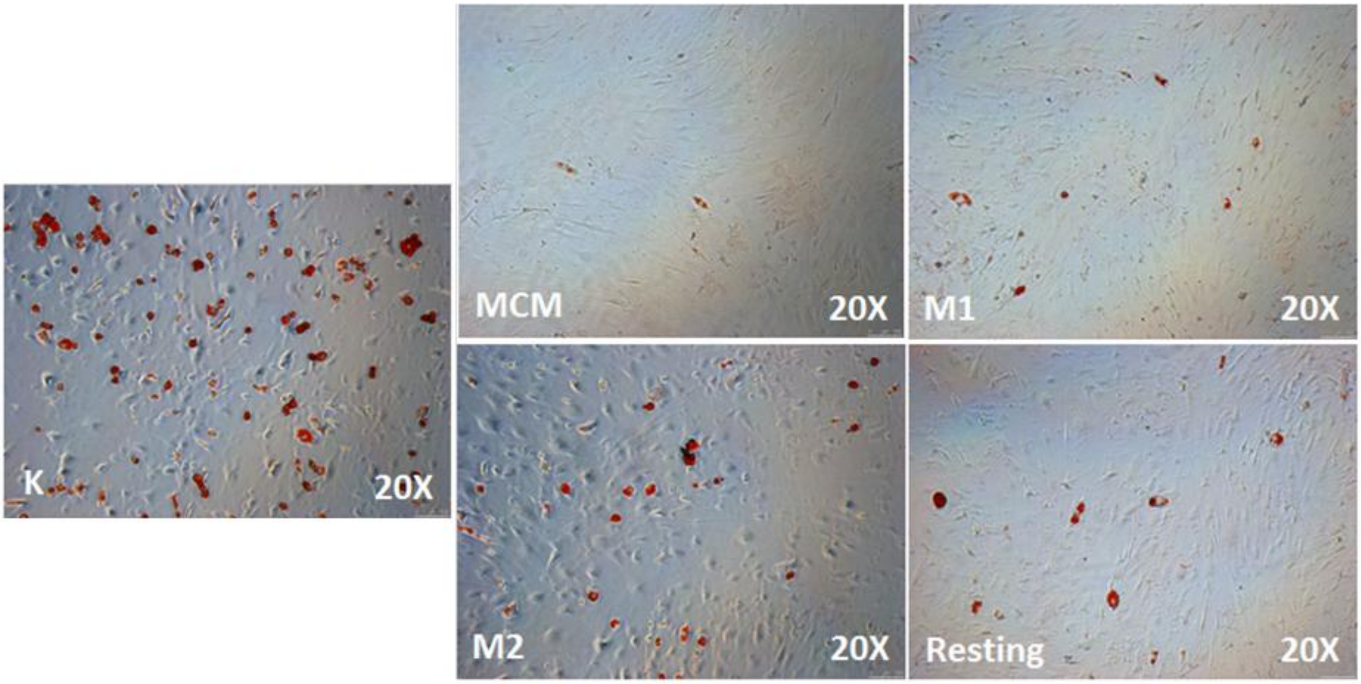

Supplementary Figure 3: Example of Oil Red-O staining performed on FAPs to mark adipocytes (stained in red).

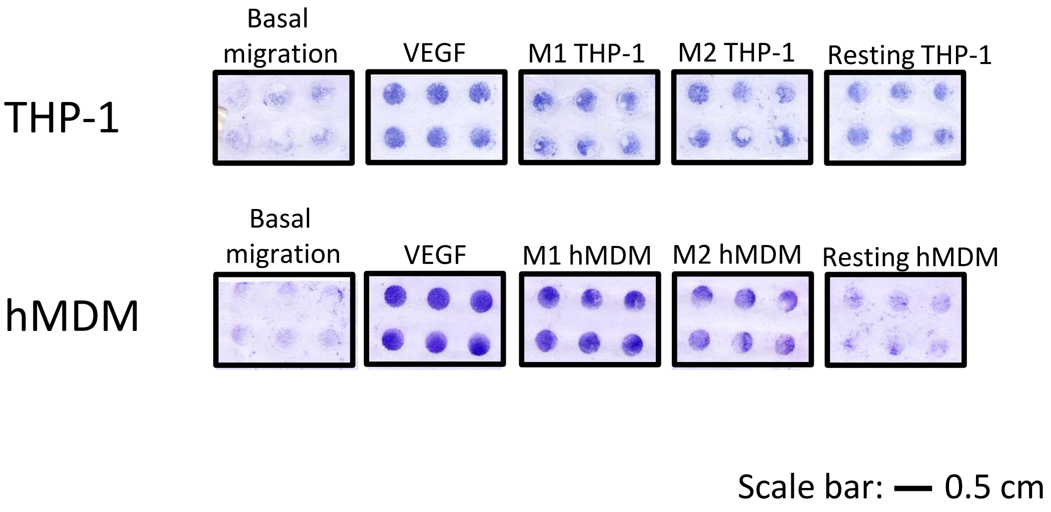

Supplementary Figure 4: Example of stained membranes obtained from the chemotaxis assays.
